# Behind the Rainbow, “Tongqi” Wives of Men Who Have Sex With Men in China: A Systematic Review

**DOI:** 10.3389/fpsyg.2019.02929

**Published:** 2020-01-14

**Authors:** Yuanyuan Wang, Amanda Wilson, Runsen Chen, Zhishan Hu, Ke Peng, Shicun Xu

**Affiliations:** ^1^Division of Psychology, Faculty of Health and Life Sciences, De Montfort University, Leicester, United Kingdom; ^2^National Clinical Research Center for Mental Disorders, Beijing Key Laboratory of Mental Disorders & Advanced Innovation Center for Human Brain Protection, Beijing Anding Hospital, Capital Medical University, Beijing, China; ^3^Faculty of Health Sciences, University of Macau, Macau, China; ^4^The George Institute for Global Health, UNSW, Sydney, NSW, Australia; ^5^School of Public Health, The University of Sydney, Sydney, NSW, Australia; ^6^Department of Population, Resources and Environment, Northeast Asian Studies College, Jilin University, Changchun, China

**Keywords:** Tongqi, China, sexual health, mental health, violence, review

## Abstract

**Background:**

Due to the restrictions and stigmatization of homosexuality in China, there has emerged the “Tongqi,” or the wives of men who have sex with men (MSM). There are around 14 million Tongqi wives whose needs for support are often overshadowed. This phenomenon has been largely under researched, this review is the first to address the current data on the Tongqi. The aim of this systematic review is to begin to provide insight into the pre-existing data and the further support that is needed for the wives of MSM.

**Methods:**

The researchers searched PubMed, Web of Science, EMBASE, PsycINFO, CNKI, Sinomed and WangFang databases from their inception date until June 7, 2019. Handsearching was also completed to provide a rich data set.

**Results:**

The articles were summarized and analyzed for thematic clusters. From the selected article, five themes emerged, including Sexual Health Issues, Intimate Partner Violence, Mental Health Status, Marriage Dissatisfaction, and Coping Strategies. These themes often intersected to provide a complex understanding of the current gaps in support provided to Tongqi.

**Conclusion:**

Tongqi wives remain a hidden population in Chinese mainstream society, who deserves a sensitive approach to support. The study revealed that the MSM wives suffer severe mental, physical, health, and life related harms. However, instead of situating them into the victim roles, many women take on an identity of empowerment and are working together, aiming to make social changes. In order to address the Tongqi phenomenon, it is also essential to reduce the discrimination toward homosexuality. Tongqi are a special group of Chinese women, they require further intensive research attention.

## Introduction

Previous international studies have focused on the mixed-orientation marriages between homosexual men and heterosexual wives (Higgins, 2002; Hernandez et al., 2011; Kissil and Itzhaky, 2015; Hopwood et al., 2019). For investigating these mixed-orientation marriages, it is important to consider the contextual environment, social, and cultural context (Kissil and Itzhaky, 2015). With the legalization of homosexual marriage in a wide range of countries, the legal marriage right for homosexual population has significantly changed. Considering the population size, China is home to the world’s largest homosexual population (Wang et al., 2019). However, homosexual marriage is not recognized by law in China (Marriage Law of the People’s Republic of China, 2001; Li et al., 2017). In China, due to discrimination, men who have sex with men (MSM) are a sexual minority and remain hidden as a population (Zhu, 2018). Consequently, Chinese MSM often marry to conceal their homosexuality and to deal with their parents’ expectation (Cheng, 2016). There is a scarcity of research focusing on Chinese women who married with gay men. Tongqi is a culturally specific neologism in China, which can be translated as “wives of gay men.” Narrowly defined, Tongqi describes heterosexual women who marry MSM, while more broadly speaking, Tongqi includes both the wives of MSM and the unmarried female partners of MSM (Liu and Tang, 2014b). Tongqi is the victimized population shadowed behind this hidden population, whom have insufficient power to make their voice be heard. With the increasing visibility of the MSM population, many wives have started to question the sexual orientation of their husbands (Zhu, 2018). This visibility has resulted in the growing number of Tongqi in China. According to a recent estimation, there is over 13.6 million Tongqi in mainland China (Cheng, 2016).

The term “marriage fraud” is used exclusively in mainland China to describe closeted MSM who pretended to be heterosexual in order to marry a non-complicit heterosexual woman (Zhu, 2018). In Chinese culture, getting married and producing offspring is a social and familial obligation (Zhou, 2006; Chen et al., 2019; Zhu et al., 2019). Thus, Chinese MSM face pressure and expectations from their parents to have grandchildren to continue the family line (Chow et al., 2011). There are also pressures on Tongqi women as well to stay married that are similar to the pressure faced by the men. Given the marriage is fraudulent the wife risks exposing the MSM husband, not only will this bring the wives shame but they will receive no compensation. There is also the unintended effect of outing the discrete MSM husband, which may create further hate toward the gay community (Zhu, 2018; Pan, 2019). In 2009, “Tongqi Family,” the first grassroot (non-official) organization for Tongqi was founded, which aims to provide psychological support and legal advice for divorce (Chow et al., 2013). Recently, increasing concerns have been raised regarding the welfare of wives of MSM (Li X. et al., 2016). Noticeably, Tongqi suffer from profound intimate partner violence (IPV, i.e., physical abuse, verbal abuse, and emotional abuse), suicidal ideation, suicide attempts, depressive symptoms, anal sex coercion, and at risk of sexual transmitted diseases (Liu and Tang, 2014a; Li X. et al., 2016; Li et al., 2017; Wu et al., 2018).

Tongqi, as a group of minority women in China, require urgent support and substantial research attention. Thus, we aimed to conduct a comprehensive review to better understand the situation of Chinese Tongqi.

## Methods

### Selection Criteria

This systematic review covers original studies on Tongqi in China. No restrictions were applied in study settings and designs due to the lack of previous research. Studies published either in English or Chinese that focused on Tongqi population were included.

### Literature Search and Data Extraction

Two researchers (RC and ZH) independently searched PubMed, Web of Science, EMBASE, PsycINFO, CNKI, Sinomed and WangFang databases from their inception date until June 7, 2019. The following key words were applied in the searching, for example, “Tongqi,” “wives of gay men,” “homowife,” “gay husband/straight wives,” “China,” “Chinese,” etc. To extract further literature, the researchers (RC and ZH) also hand-searched the reference lists of review papers and articles. Two researchers (ZH and YW) independently screened the relevant articles, and another two researchers (RC and YY) independently extracted data. To reach agreement, any inconsistencies during screen and data extraction were resolved through discussion between the researchers. The following information was extracted from the included articles: first author with publication year, province, study setting, study design, recruitment method, sample size, demographic information of participants, assessed aspect of the participants, main findings, and marriage related information.

## Results

### Study Characteristics

**FIGURE 1:**
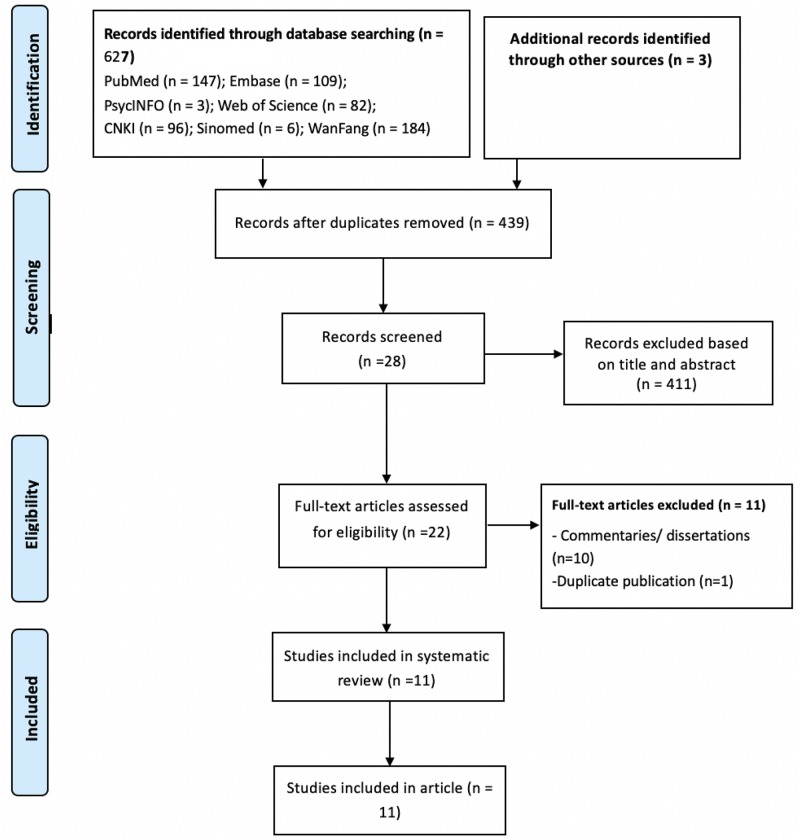
PRISMA flowchart.

From the database and handsearching 627 articles were initially identified and 11 studies (Tang and Zhang, 2013; Liu and Tang, 2014a; Tang and Liu, 2014; Tang and Yu, 2014; Zhang et al., 2014, 2015; Li X. et al., 2016; Li X. H. et al., 2016; Li et al., 2017; Wu et al., 2018; Yan et al., 2019) met the selection criteria and were selected for the review (see Figure 1).

### Summary of Main Findings Across Studies

Based on the selected article, five themes emerged, this included Sexual Health Issues, Intimate Partner Violence, Mental Health Status, Marriage Dissatisfaction, and Coping Strategies.

### Sexual Health Issues

Tongqi in China are a high-risk population of HIV and sexual transmitted diseases. For instance, Li et al. (2017) reported that in a sample of 144 Chinese gay wives, less than 30% had undergone HIV screening and 5.6% were HIV-positive; furthermore 35.3% had sexually transmitted infections (STIs). The literature was inconsistent with reporting wives’ use of condoms during sexual intercourse with their husband. Li et al. (2017) found that 60% of the wives would use condoms during intercourse with their husbands. Li X. et al. (2016) found that 40.2% never used condoms with their husbands, and 47.6% of these wives who never used condoms were at high risk of contracting HIV.

### Intimate Partner Violence

Tongqi in the articles reviewed frequently suffered from IPV. As reported by Zhang et al. (2015), 33% Tongqi reported being verbally and/or physically abused by their husbands, with 22% reporting suffering from physical abuse on more than one occasion. In addition, Tongqi also suffered from family cold violence. Family cold violence refers to the indifferent behaviors in conflicts and arguments, such as silence, sarcasm, stopping sex, insult, and other mentally hurtful informal violent behaviors. Zhang et al. (2015) also found that 74.2% Tongqi reported that they suffered from long-term indifference with their partners, and their husbands reviled 30.5% of them excessively. More recently, using a sample of 195 Chinese Yan et al. (2019) developed a 10-item scale to measure family cold violence. The higher the total score on the scale reflects more frequent family cold violence and indicates there is a severity of suffering from family cold violence. The research results found that most of Tongqi received a high total score measured by this scale.

### Mental Health Status

Tongqi were at high risk of mental ill health in the studies reviewed. For example, Li X. et al. (2016) found that once the wives knew that their husbands were gay, 59.8% Tongqi had strong suicidal ideation, and 10% of them went on to attempt suicide. The majority (90%) of Tongqi also showed depressive symptoms. Liu and Tang (2014a) conducted a qualitative study with using a sample of eight Tongqi aged from 27 to 35. Those participants discussed that they experienced suffering from insomnia, depression, and revealed that they had attempted suicide. Another survey including 178 Chinese Tongqi showed that 61.2% had thought about suicide, with 11.8% attempting suicide (Wu et al., 2018).

### Marital Dissatisfaction

There was very little satisfaction found from Tongqi wives, they expressed desperation in their marriages and had overall a low marital satisfaction (Tang and Zhang, 2013; Tang and Liu, 2014; Tang and Yu, 2014). Marital dissatisfaction was reported in aspects of husband’s deception (true sexual orientation and homosexual affaires) and their own personal sexual gratification. In terms of disclosure of their true sexual orientation toward men, less than 9% of the husbands had disclosed their MSM orientation initially, 40% Tongqi found out that their husbands were homosexual after 3 years, and most wives discovered their husbands’ true sexual orientation through their mobile internet activities (Zhang et al., 2014). In terms of MSM affairs, 17% caught their husbands in the act of having sex with other men. Li et al. (2017) found that 44.2% stopped having sex after discovering their husbands were MSM. In addition to a lack of sexual satisfaction, 9.9% said that they suffered from sexual abuse, and 62% reported feeling depressed due to abstaining from sexual intercourse with their husband. In addition, Tang and Yu (2014) reported that some Tongqi wives had never initiated physical intimate contact with their husbands, nor did they engage in sexual intercourse.

After knowing the truth, those who chose to divorce tended to be a younger age. The older in age, the more likely the wives were to decide to maintain the marriage and continue to endure (Li X. et al., 2016). The literature identified the following reasons for Tongqi to maintain marriage including concerns about children, mutual properties, economic concerns, parental pressure to avoid divorce, worry about finding new partners, reputation, affection for husband, and pressure from parents and responsibilities. Zhang et al. (2014) reported that the most common reason not to divorce was concerns about children and mutual properties. According to Liu and Tang’s (2014a) qualitative study with eight Tongqi, participants said that they still had strong and loving feelings toward their husbands. The participants were also concerned that it would be difficult to find a new partner after breaking-up. Out of the eight, five participants said they prefer divorce but could not deal with the pressure from multiple sources such as parents, finance, social opinion, and family responsibilities.

### Coping Strategies

In order to overcome their unsatisfactory marriage, Tongqi applied different coping strategies, including turning to religion, joining online support groups, and having extramarital sex partners. In Liu and Tang’s (2014a) study, three participants said that they placed their hope with their religion and relied on their spiritual sustenance. Liu and Tang (2014a) also reported that some participants joined online Tongqi wife support groups in order to have a place where they could receive and give mutual encouragement to be positive about life. Amongst wives who maintained their marriage, 30% had extramarital sex partners largely because of their unsatisfactory sex life and in order to rebuild confidence and to vent out discontent about their husbands (Li et al., 2017). Noticeably, Wu et al. (2018) found that the coping style has important influence on Tongqi’s life including their mental status. The coping style of the MSM wives had a full mediating effect on the association between IPV and suicidal ideation and a partial mediating effect on suicide attempts (Wu et al., 2018). However, the self-invented coping strategies were not sufficient for this group. Li X. et al. (2016) conducted a qualitative study with a sample of eight tongqi aged from 18 to 75 years old. Most of the participants said that they required professional psychological intervention to cope with the stress that came with being a Tongqi wife.

## Discussion

This is the first review to target the hidden population “Tongqi” in China, and provided a comprehensive overview of the existing research. The current study can be considered as a cornerstone for the future research in this area. From the literature reviewed five themes, discussed in depth above, were generated. To summarize these themes were, Sexual Health Issues, Intimate Partner Violence, Mental Health Status, Marriage Dissatisfaction, and Coping Strategies. The study revealed that Chinese Tongqi suffer severe mental, physical, health, and life related harms.

The Tongqi phenomenon in China is embedded within the Chinese socio-cultural context. Compared with western developed countries, discrimination toward the homosexual population is still relatively more severe in China (Wang et al., 2019). Like other cultures, the Chinese environment tends to push women into marriage. Chinese women who are delaying marriage are called “leftover women” on social media, or unmarried women that no one want (Murti, 2019). Due to the lack of laws concerning homosexual marriage and the pressure on MSM in China to continue the family line, it is more likely women enter into marriage without carefully considerations, which could consequently increase the population of Tongqi.

The result revealed that Tongqi are at risk of STIs, including HIV. Amongst the Chinese HIV infected population, MSM were the group had the highest risk of new infection and undiagnosed infection (Wu et al., 2015) as unprotected sexual activity in MSM is one of the major routes for transmission in the Chinese population (Chow et al., 2013). MSM then tended to have unprotected sexual intercourse with their wives (Wu et al., 2018). Wives of MSM are at high risk for HIV because they were often unaware that their husband had extra marital affairs and therefore perceived that they were at low risk of contraction (Solomon et al., 2010). In China, between spouse and any regular sexual partner, the use of a condom implies a lack of fidelity or lack of mutual trust (Li X. et al., 2016). Moreover, unprotected sex is considered to be a normal part of heterosexual marriage (Ren and Yuan, 2018). In order to reduce the risk of transmission amongst the Tongqi population, previous researchers have recommended that MSM should be provided knowledge about the potential threats of HIV/STI transmission to wives (Pandya et al., 2012). However, to date there are little to no knowledge based Chinese interventions to support MSM to reduce their risk of transmission.

The results showed that Tongqi women have various susceptibilities to serious mental health disorders, such as suicide. Over half of the women felt suicidal with 10% attempted suicide, they also reported high rates of insomnia and depression (90%). Suicide is considered taboo within Chinese culture; with the stigma attached to suicide it is even shameful to admit suicidal behaviors. It is debated whether men or women have a higher suicide rate in China, but like the Tongqi wives in this review, women are more likely than men to have suicidal ideations and attempts (Cao et al., 2015). Those wives who participated in anal sex were 7.8% more likely to have suicidal thoughts than those who did not. In general, Tongqi women had little support to cope with suicidal ideations (Li X. H. et al., 2016). The qualitative studies in this review also suggested the Tongqi suffered from insomnia. This supports findings from previous research, which suggest women have higher rates of insomnia in China (Cao X. L. et al., 2017; Tang et al., 2017) and that there is a co-morbidity of insomnia, anxiety, and depression (Chen et al., 2018). Rate of marital wellbeing also suggests there is an association between, being depressed and husbands’ unhappiness. Furthermore, husband’s commitment and instability were also key indicators of depression, even when interpersonal factors and risk factors were controlled for Cao H. et al. (2017). Depression has been known to co-occur with IPV, if a Chinese woman is a victim of violence from an intimate partner she is more likely to exhibit depression. Depression and low self-esteem have been linked in China to predicting IPV (Lin et al., 2018). In the current review, it is unknown whether the Tongqi wives were depressed before IPV and there is no mention of low self-esteem in the literature reviewed.

Tongqi women were at risk of IPV; this included verbal and physical abuse from their husband to cold family violence from the extended family and in-laws. According to a scoping review, the rates of violence, as reported by Tongqi wives, is higher than the national rate of IPV against Chinese women who are not married to MSM. In the general population, the review reports that at the most 24.5% of Chinese women faced psychological abuse, at most 5.5% physical violence, and 0.3–1.7% sexual violence (Yang et al., 2019). In this review Tongqi women reported, 22% reported being a victim of physical violence, 16.5% more than the general population of women. While the rates of physical violence and psychological violence were combined at 33%, this is 3% higher than the rates for general Chinese women. Even though sexual violence was reported in the reviewed literature the rates of sexual violence are unknown, future research should seek to find out the rate of sexual violence and separate each type of violence when reporting rates. MSM who have sex with women are also at a higher rate than MSM to be victims of violence, for example being thrown objects at and threats to reveal their sex with men to other people. This review did not provide insight into the gendered symmetry of IPV (Chen and Chan, 2019) amongst Tongqi wives and their husbands; this area also needs further exploration. Cold family violence was a result of being a Tongqi wife, in this review, who wanted to file for divorce. There is little to no literature around family cold violence, not only from the wives family but also from the husbands family, however, research on domestic violence and traditional values may explain the Tongqi wives experience. Research suggests that the more gender traditional beliefs a person has the more likely they are to be a perpetrator of violence (Cheung and Choi, 2016). How cold violence is experienced by Tongqi wives remain unknown.

In the literature reviewed various coping strategies were reported. This included both maladaptive and positive coping styles. Maladaptive coping included extramarital sex partners, potentially increasing the risk of transmission of HIV to a larger general population via the Tongqi wife who unknowingly contracted HIV from her husband. In a qualitative study, men who have sex with men and women (MSMW) were interviewed and the findings support that men are reluctant to use condoms with their female partner even if diagnosed with HIV due to the stigma attached to having HIV in China. They also were less likely to recommend their female partner be tested for HIV (Wang et al., 2015). Positive coping included online support group and several women interviewed stated a need for professional psychological support. When interviewed Chinese service users preferred mental health services not within their neighborhood but believed community services were best placed on the Internet (Chen, 2018). Online self-help could be a cost-effective way to deliver mental health services in China due to the shortage of mental health professionals, however this needs to be provided in a guided manner (Chen and Zhu, 2016), unlike the Tongqi online community that currently has no guided mental health support, just peer support. A guided community online support system could be delivered by testing a psychological intervention within community to better support Tongqi with their psychiatric needs.

The results showed that although Tongqi suffered both from and during MSM marriage, there were larger concerns made them to stay in the marriage. According to the Marriage law of China, if the wife or husband are unfaithful with a third party of the opposite sex during marriage, the non-fault party has the right to ask for compensation at the time of divorce (Marriage Law of the People’s Republic of China, 2001). The appeals for divorce by the Tongqi wives are frequently rejected by the court, the law of third party only recognizes the opposite sex not the MSM, who are having extra marital affairs with the same sex (Li X. et al., 2016). Thus, to some extent, the Tongqi’s rights to compensation are not protected by Chinese marriage law and could be largely affecting their decisions to divorce or stay in the marriage. It is important then for policy makers to consider and protect Tongqi’s rights in marriage and divorce to provide more women the option to leave a dissatisfied marriage.

Tongqi remain a hidden population in the Chinese mainstream society. Although Chinese media have started to report news on Tongqi, the media prefer to use eye-catching headlines which emphasize the lack of sexual satisfaction in marriage, for example, “my husband never saw me naked” or “still a virgin in her 60s”(Zhu, 2018). It is an important step to improve the media’s presentation and instead focus on the Tongqi phenomenon as a social issue, and likewise social media should report on this group in a more respectful way. Public attention should focus on Tongqi from a comprehensive view, rather than the sexual unsatisfaction as unwanted wives. In addition, Tongqi is a group that has been victims of extra marital affairs and they have suffered from various stressors. However, instead of situating them into the victim roles, many women take on an identity of empowerment and are working together, aiming to make social changes. For example, they have proposed to change the marriage law to allow for consideration that their MSM spouse is at fault, they disclose their Tongqi identity to alert other women but also in an attempt to de-stigmatize the identity, and most importantly they are campaigning for same-sex marriage to give MSM the right to marry their male partners. In order to address the Tongqi phenomenon, it is also essential to reduce the discrimination toward homosexuality. China’s one-child policy pressures homosexuals to enter marriage (Li X. et al., 2016). It is hard for Chinese MSM to find a balance between being gay and fulfilling duties of passing the family line (Steward et al., 2013).

The current review has several limitations. First, due to the availability of the existing research, we opted to summarize the information instead of synthesizing the data into a meta-analysis, which can create a larger, generalizable effect size. Second, the majority of the included studies were in Chinese rather than English, suggesting that the west has placed little focus on Tongqi wives, it is truly an under-research area. Third, due to the data used, we were unable to provide practical suggestions for Tongqi to improve their life conditions. Finally, we aimed to reveal the Chinese Tongqi phenomenon within the specific Chinese cultural context, future research should look to comprehensively compare wives of gay men from different socio-cultural environments. For example, in the United States, there is a cultural phenomenon called the “down low,” these are MSM who marry heterosexual women. The wives of “down low” men have historically felt forced to stay in the marriage, similar to the Tongqi, but there are differences in that “down low” women have more opportunity for financial freedom and legal protection. Furthermore, the term “down low” women has been largely black women and black women in the United States have the lowest rates of marriage (Glenn and Spieldenner, 2013), unlike the Tongqi. Similarities do exist though in the risk of HIV transmission from “down low” husbands (Harawa et al., 2006; Sandfort and Dodge, 2008; Bond et al., 2009). Also, much like in China, it is argued that in the United States “the down low” phenomena has a cultural and community based context (Barnshaw and Letukas, 2010). The same is true in India, while there is a push for MSM to marry and have children, however, the majority of MSM remain single (Solomon et al., 2016). Here too the rates of HIV infection are higher than in the out, non-MSM community (Solomon et al., 2016). Finally, Israel has Jewish men who are married and also identify as gay but little research has been done on their wives’ mental and physical health (Zack and Ben-Ari, 2019) to inform the research on the Tongqi. In conclusion, Tongqi are a special group of Chinese women, which requires further intensive research attention, just like in many other low/middle-income countries where homosexuality is not recognized by law. In order to address the Tongqi phenomenon, it is also essential to reduce the discrimination toward homosexuality in China. This would provide space for an alternative lifestyle such as choosing not to marry (like in India) and being provided the opportunity to marry whomever one wants (as allowed by law in the United States).

## Author Contributions

YW and RC: protocol design, search, screening and assessment of studies, data extraction, data analysis and interpretation, and writing the manuscript. AW: data extraction, data analysis and interpretation, and writing the manuscript. ZH: protocol design, search, and screening and assessment of studies. KP: protocol design, screening and assessment of studies, data extraction, and data analysis. SX: data interpretation, data analysis and interpretation, supervision of the review, writing, and reviewing the final manuscript.

## Conflict of Interest

The authors declare that the research was conducted in the absence of any commercial or financial relationships that could be construed as a potential conflict of interest.
